# Increased CH_4_ Oxidation in Arctic Tundra Ecosystems Caused by Vegetation‐Mediated Soil Drying

**DOI:** 10.1111/gcb.70810

**Published:** 2026-03-26

**Authors:** Mats P. Björkman, Jan Dietrich, Mabel L. Gray, Argus Pesqueda, Mario Rudner, Laura Rasmussen, Joel D. White, Bo Elberling, Robert G. Björk

**Affiliations:** ^1^ Department of Biological and Environmental Sciences University of Gothenburg Gothenburg Sweden; ^2^ Gothenburg Global Biodiversity Centre Gothenburg Sweden; ^3^ Department of Ecology and Environmental Science University of Umeå Umeå Sweden; ^4^ Department of Earth and Planetary Sciences American Museum of Natural History New York New York USA; ^5^ Center for Ecological Research and Forestry Applications, Edifici C Universitat Autònoma de Barcelona Barcelona Spain; ^6^ Department of Earth Sciences University of Gothenburg Gothenburg Sweden; ^7^ Copenhagen Data Lab, Department of Mathematical Sciences University of Copenhagen Copenhagen Denmark; ^8^ Department of Geoscience University of Tromsø Tromsø Norway; ^9^ Department of Geosciences and Natural Resource Management Copenhagen Denmark

**Keywords:** Arctic, international tundra experiment, methane oxidation, open‐top chambers, tundra, vegetation change

## Abstract

Arctic tundra soils can act as an important sink for atmospheric methane (CH_4_). However, the role and magnitude of this process, and how it will change during future climate scenarios, are poorly understood. The vegetation is changing with a warmer Arctic climate, with taller plants, more shrubs, and altered vegetation patterns. These changes are predicted to be strongest in moist to wet regions, areas usually associated with CH_4_ production. Additionally, these changes in growth patterns can increase evapotranspiration rates, leading to enhanced soil aeration, favouring CH_4_ oxidation. Here, we investigate CH_4_ dynamics within long‐term (> 25 years) passive air warming treatments, using five plant communities with contrasting soil moisture and nutrient regimes. These treatments reveal a strong increase in atmospheric CH_4_ oxidation in two dry ecosystems (140.4% ± 8.1% and 204.2% ± 19.3% for a Dry Heath and Dry Meadow, respectively), and a strong reduction of CH_4_ emissions (91.2% ± 18.6%) in a Tussock Tundra community. In contrast, our investigation of Mesic and Wet Meadows showed no significant treatment effects, with only limited CH_4_ exchange in the Wet Meadow. Furthermore, when inhibiting CH_4_ oxidation in the surface soil, we found evidence of CH_4_ production even at the driest site (Dry Heath), indicating a potential for CH_4_ production throughout the landscape. Although soil temperature and moisture have been put forward as strong regulators of CH_4_ fluxes, they did not consistently explain our observed changes. Instead, we argue for interactions between vegetation change and near‐surface soil characteristics. The observed shift in plant composition and increased vegetation height, along with warmer air temperatures, enhanced evapotranspiration and surface soil aeration, thereby stimulating methanotrophy and leading to increased CH_4_ oxidation. This vegetation‐induced climate feedback would aid the predicted temperature‐dependent increase of CH_4_ oxidation in the Arctic, potentially mediating CH_4_ emissions from the region.

## Graphical abstract

In a warmer Arctic, vegetation changes can play a critical role in methane dynamics. Our results indicate that taller vegetation, shifts in species composition, and warmer air temperatures enhance soil aeration by increasing evapotranspiration, thereby promoting methane oxidation in surface soils. This results in an increased uptake of atmospheric methane in drier ecosystems, while moderating emissions from wetter ones. These vegetation‐driven responses, although not uniform among the plant communities investigated, might then mitigate the large methane emissions estimated from the region.

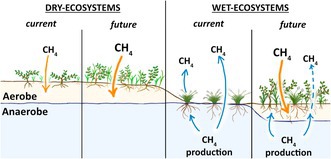

## 1 Introduction

It is well established that the concentration of the powerful greenhouse gas methane (CH_4_) is increasing in the atmosphere (Parmentier et al. 2024). Although global anthropogenic CH_4_ emissions are estimated to represent only around 3% of the global anthropogenic greenhouse gas emissions, the increase in atmospheric CH_4_ concentrations has significantly contributed, ∼23% (∼0.62 W m^−2^), to the additional radiative forcing accumulated in the lower atmosphere since 1750 (Etminan et al. 2016). While sources and their relative contribution to the CH_4_ budget are relatively well understood, mechanisms governing CH_4_ consumption from the atmosphere, especially within soils, are poorly constrained (Voigt et al. 2023).

Growing evidence shows a significant and widespread sink of atmospheric CH_4_ in the Arctic landscape (Curry 2007; Juncher Jørgensen et al. 2015; Lau et al. 2015), associated with active oxidation of CH_4_ by methanotrophic bacteria in upland mineral soils (Stackhouse et al. 2017). However, this sink can also be the main regulating process within wetland ecosystems (Le Mer and Roger 2001). The Arctic upland oxidation of atmospheric CH_4_ has recently been shown to play a major part in future climate scenarios (Oh et al. 2020). When accounting for CH_4_ oxidation in the model, the projected climate‐driven doubling of the region's CH_4_ emissions (Koven et al. 2011; Lawrence et al. 2015; Schuur et al. 2013) was mitigated to a final increase of only 18% (Oh et al. 2020). At low temperatures, soil CH_4_ oxidation can match or exceed CH_4_ production, but across broader warmer ranges, CH_4_ production typically shows equal or greater temperature sensitivity than oxidation (Zheng et al. 2018). Process‐based modelling indicates that the strong influence of CH_4_ oxidation on net CH_4_ fluxes is explained by differences in temperature sensitivity between high‐affinity methanotrophs (Bacteria oxidising CH_4_ at atmospheric concentration), and methanogens (Archaea producing CH_4_). In these models, methanotrophic activity often exhibits a more rapid response to changes at low temperatures than methanogenic production, thereby partially offsetting the predicted increase in CH_4_ emissions under a warmer climate (Oh et al. 2020, 2016). While methanogenesis mainly occurs under anoxic conditions in water‐saturated soils (e.g., wetlands, fens, water‐logged soil and anoxic microsites), methanotrophic activity is more pronounced in aerated/dry soils or regions with a relatively high oxygen level (e.g., rhizosphere, above or close to the water table) (Le Mer and Roger 2001). This generalized picture is, however, not completely accurate, with clear evidence of methanotrophy observed during anaerobic conditions (Thauer and Shima 2008) and methanogenesis in aerobic (Angle et al. 2017). Both these processes depend on the soil water content in Arctic systems (Lawrence et al. 2015; Stackhouse et al. 2017; Torn and Chapin 1993), as this regulates the diffusion of gases and the availability of both CH_4_ and oxygen in the soil, thus influencing the redox potential. The soil moisture content in surface soils, however, is mainly regulated by evapotranspiration (Zona et al. 2023), with temperature, wind exposure, and vegetation cover playing major roles (Aalto et al. 2013), along with local drainage conditions. These factors collectively impact the availability of CH_4_ and oxygen in the soil, thereby influencing the interplay between methanogenesis and methanotrophy in Arctic soils.

It is well‐established that a warmer climate leads to changing vegetation patterns in the Arctic (Bjorkman et al. 2018; Elmendorf, Henry, Hollister, et al. 2012; Pearson et al. 2013). Empirical data from monitoring and long‐term warming experiments point towards an overall taller plant community (Bjorkman et al. 2018), and an increase in biomass (Hudson and Henry 2009), especially in moist and wet ecosystems (Elmendorf, Henry, Hollister, et al. 2012). In addition, an expansion of shrubs has been observed (Myers‐Smith et al. 2011; Sturm et al. 2001) with expansion being more pronounced for evergreen shrubs in dry ecosystems and deciduous shrubs in wet ecosystems (Scharn et al. 2022) and with co‐occurring changes in root distribution and soil aeration (Angers and Caron 1998; Keuschnig et al. 2022). Furthermore, a reduction in plants with aerenchyma tissue, which can facilitate gas exchange (including CH_4_ and oxygen) between soil and atmosphere (Joabsson et al. 1999; Ström et al. 2012), has also been observed at some sites (Hollister et al. 2005; Kwon et al. 2016; Scharn et al. 2021), negatively influencing the magnitude of CH_4_ emissions. Although the direction towards taller Arctic plant communities, shrub expansions and changes in species composition are well‐established (Bjorkman et al. 2018; Elmendorf, Henry, Hollister, et al. 2012; Myers‐Smith et al. 2011), the secondary effect on CH_4_ fluxes, especially CH_4_ oxidation, is understudied (Dean et al. 2018). This knowledge is fundamental for understanding current and future vegetation‐soil‐atmosphere feedback in the Arctic.

In this study, we used a long‐term passive air warming experiment with pronounced changes in vegetation composition (Scharn et al. 2022, 2021), plant traits (Baruah et al. 2017), and loss of aerenchymatous plants (Keuschnig et al. 2022; Molau 2010) to investigate how a projected future vegetation regime, in line with the year 2050–2080 (Sarneel et al. 2020), will influence the ecosystem's CH_4_ dynamics. We hypothesized that the long‐term passive air warming will influence the in situ fluxes with higher CH_4_ oxidation rates within the treatment as a result of increased soil temperature (Oh et al. 2020). In addition, we hypothesized that a reduced soil moisture content in the warming treatment (Scharn et al. 2021) will significantly increase CH_4_ oxidation rates in the soil, enhancing the uptake rates of atmospheric CH_4_ within drier ecosystems and reducing net CH_4_ emission rates in wetter plant communities.

## 2 Methods

### 2.1 Site Description

This study was conducted at the Latnjajaure Field Station (LFS, 68°20′ N, 18°30′ E and 981 m a.s.l.), in the northern part of Sweden, which is situated within the U‐shaped valley Latnjavággi. The climate here has a mean annual temperature ranging from −1°C to −3°C, with February being the coldest month (−9.4°C ± 5.2°C) and July the warmest (8.6°C ± 4.1°C), and an annual precipitation ranging from 600 to 1100 mm (LFS monitoring data, 1992–2018, Scharn et al. 2022). The climate is typical of the Oro‐Arctic (Virtanen et al. 2016) with prevailing winter conditions (September to end of May), where the accumulated snow is a major water source for many of the plant communities during the growing season.

Since the early 1990s, LFS have been a master site within the International Tundra Experiment (ITEX, Henry et al. 2022; Henry and Molau 1997) with open‐top chambers (OTC) used as a passive air warming treatment, increasing the interior air temperatures by 1°C to 3°C (Hollister et al. 2023; Marion et al. 1997; Molau and Mølgaard 1996). Five OTCs and five control plots (hereafter referred to as “*OTC*” and “*Ambient*” treatment, respectively) were established within five different plant communities during 1993 and 1994, with the OTCs deployed year‐round. Hence, the OTC are designed to manipulate the air temperatures around the plants, while minimising effects on humidity, precipitation, access for pollinators and herbivores, etc. (Molau and Mølgaard 1996). Other edaphic factors, such as snow conditions, soil moisture and soil temperatures, tend to be influenced to various degrees at different study sites (for more details see Hollister et al. 2023). The five plant communities include two dry sites: Dry Heath (HD, Molau 1997; Molau and Alatalo 1998), Dry Meadow (MD, Molau 2001), and three mesic to wet sites: Mesic Meadow (MM, Stenström and Jónsdóttir 1997; Stenström et al. 1997), Wet Meadow (MW, Molau 2001) and Tussock Tundra (TT, Keuschnig et al. 2022; Molau 2010; Molau and Shaver 1997). The plant communities differ mainly in terms of their dominant plant species. HD: *Betula nana* , *Cassiope tetragona* , *Salix herbacea* and *Empetrum nigrum* , MD: *Dryas octopetala* , *Carex bigelowii* , *Salix reticulata* , and *Bistorta vivipara* , MM: *D. octopetala* , *Carex vaginata* , *B. vivipara* and *E. nigrum*. MW: *B. vivipara*, *C. bigelowii* , *Calamagrostis stricta* , and *Poa arctica* , and TT: *Eriophorum vaginatum* , *Phyllodoce caerulea* , *Vaccinium vitis‐idaea* , and *S. herbacea* . Soil pH and moisture content are also contrasting factors. The Meadow communities have a relatively alkaline and nutrient‐rich soil (Molau and Alatalo 1998), while the Heath community is found on a more acidic, glacial moraine. Further site description and site name nomenclature can be found in (Sarneel et al. 2020; Scharn et al. 2022, 2021).

As the OTCs are left continuously in the field, the setup has the potential to influence the snow cover and duration inside the chamber compared to the surrounding conditions (Hollister et al. 2023). At LFS, the snow depth varies among the communities, commonly deeper than the OTCs at TT (approx. 60–100 cm) (Keuschnig et al. 2022) and MW (approx. 60–100 cm), while lower at HD (approx. 20–60 cm), MM (approx. 10–50 cm) (Björkman et al. 2010), and MD (approx. 10–30 cm), with some additional snow in the OTCs (Scharn et al. 2022). The snow duration also differs among the plant communities, where the OTCs in communities with a thin snowpack (MD, MM, and HD) typically melt out earlier (Lindblad 2007).

### 2.2 CH_4_ Flux Measurements

Net fluxes of CH_4_ (*F*_net_) were measured on a bi‐weekly basis during the growing season of 2017 and 2018 (June—August). A transparent chamber (Ø = 20 cm, h = 20 cm) equipped with a pressure vent was fitted to pre‐installed soil collars (inserted 5–10 cm into the soil) and an airtight seal was obtained by folding down a rubber tape from the chamber over the collar. The corresponding change in CH_4_ concentration in the chamber was measured using an Ultraportable Greenhouse Gas Analyzer (operating at 1 Hz, UGGA, Los Gatos Research, San Jose, U.S.). Measurements were performed manually with a chamber closure time of 4 min and a minimum ventilation time of 1 min between each measurement. Three measurements were performed per collar and measuring occasion to generate an average *F*_net_. Fluxes were determined as a linear regression between CH_4_ concentration and time and adjusted for elevation and chamber temperature. In addition, the first 30 s of the measurements were removed to account for disturbance.

Although the ITEX set‐up was initiated to study vegetation responses to warmer air temperatures, hence no *F*_net_ measurements were done before the start in 1993 and 1994, we have included flux data from an additional survey of ambient conditions, *F*_2022_ (bi‐weekly measurements from June to August 2022), from two of the plant communities: MD (12 plots) and TT (12 plots), as well as earlier published ambient data, *F*_Keuschnig_, from TT (Keuschnig et al. 2022).

The measured *F*_net_ is a result of the combined soil production (*P*_soil_) and soil consumption, the latter being composed of the oxidation of soil‐derived (*C*_soil_) as well as atmospheric (*C*_atm_) CH_4_. On occasions when production and consumption rates are equal, this would generate a net *F*_net_ of zero. To include valid low or zero flux estimates, all CH_4_ flux data were included after an initial quality check, with outliers removed (three times the standard deviation, *σ*) for data in the range; 0 < *R* ^2^ < 0.8, while all data with an *R* ^2^ > 0.8 were included (Figure S1).

To evaluate relationships between soil temperature (see description below) and net soil uptake of CH_4_ (*F*_net_<zero), temperature sensitivity coefficients (Q_10_) were obtained for each plant community and treatment based on the Arrhenius equation by plotting the natural logarithm of the in situ CH_4_ uptake rates against soil temperature (in 1000/K, Davidson and Janssens 2006).

### 2.3 CH_4_ Oxidation Inhibition

An additional CH_4_ oxidation inhibition experiment was performed to evaluate *P*_soil_ at all plots in July 2019, using acetylene (C_2_H_2_) as a specific methanotrophy inhibitor (Ding et al. 2004; King 1996; Pedersen et al. 2018). To generate acetylene, 1.23 g calcium carbide (CaC_2_) was placed in a cup within the collar, and the collar was then covered with an opaque chamber (h = 10 cm), followed by water addition through a rubber septum into the cup (using a 10 mL syringe and needle). The following reaction generated an acetylene concentration of approximately 10% vol in the chamber. Each plot was incubated for 2 h (Pedersen et al. 2018). Before and after the incubation, CH_4_ fluxes were measured following the outline of 2017 and 2018, using an LI‐7810 trace gas analyser (LI‐COR Biosciences, Lincoln, U.S.). If all methanotrophic processes were inhibited by the acetylene, the flux measurement following treatment should give the true *P*_soil_, however, our incubation experiment did not facilitate any means of checking that the inhibition was complete, and the measured *P*_soil_ should be seen as a lower indication of the potential *P*_soil_. To facilitate calculations of the CH_4_ oxidation rates, the following relationship was assumed: *F*_net_ = *P*_soil_—*C*_soil_—*C*_atm_, and that *C*_soil_ = *P*_soil_ when the total oxidation is equal to or larger than the *P*_soil_. To ensure true *P*_soil_ values, an *R* ^2^ cutoff at 0.8 was inferred on the post‐incubation measurements, while no cutoff was used for the initial *F*_net_ measurements.

### 2.4 Soil and Vegetation

In connection with all flux measurements, soil temperatures at—5 cm (*T*_soil_) and soil water content at 0–6 cm (*SWC*) were recorded at four locations around the chamber (using a hand‐held thermometer and an ML3 ThetaProbe, Delta‐T Devices, Cambridge, U.K., respectively), giving an average per collar and occasion. To complement the temperature and soil moisture data, additional TOMST loggers (TMS Standard, Praha, Czech Republic) were installed in two *OTC*s and two *Ambient* plots per plant community in 2020 (Data period analysed 1st June 2020 to 31st August 2024), recording temperatures at −8, 2 and 15 cm (*T*_*soil_TMS*_, *T*_*surface_TMS*_ and *T*_*air_TMS*_, respectively) and soil moisture (*SWC_*_*TMS*_) integrated over 0 to −12 cm (Wild et al. 2019). This data was also used to identify the day of snow melt for each plant community according to Man et al. (2023), where the day of snow melt was set to the first day of three consecutive days without snow after 1st of April each year.

Additional soil data were also collected, including pH, soil organic matter (SOM), and carbon and nitrogen ratio (C/N‐ratio) from 5 cm deep soil cores (Ø = 2.5 cm) collected in July 2019 (Table 1). General canopy heights (*z*_canopy_) were investigated in 2020 by laying out two lines, from corner to corner in plots, forming a cross with nine marked investigation points, where the height from ground to the top of the canopy was recorded. For vegetation analysis, the plot‐level ITEX point‐frame monitoring data from 2016 (Table 1, Scharn et al. 2022) were included for present coverage of functional types, including graminoid (*%*_Gram._), forbs (*%*_Forbs_), evergreen shrubs (*%*_E‐shrub_), deciduous shrubs (*%*_D‐shrubs_), aerenchyma plants (*%*_Aeren._, including *C. bigelowii* , *E. angustifolium* , and *E. vaginatum* ), as well as for the species *E. vaginatum* (*%*_Erioph._) separately, due to its dominance in TT. The above‐ground biomass of the vascular plant community (*m*_Bio_) was calculated using a non‐destructive approach by combining the point‐frame data with plant trait measurements (collected in 2016 and 2020, respectively) and the pre‐established biomass relationships (Molau 2010). Also, data from an analysis of the initial decomposition rates (*k*_TBI_) and stabilization rates (*S*_TBI_) following the Tea‐bag Index (Table 1, Sarneel et al. 2020) were included to check additional explanatory parameters.

**TABLE 1:** Summary of soil and vegetation characteristics used in the modelling, including soil organic matter (SOM), pH, carbon and nitrogen ratio (C/N), the stabilization rate (*S*_TBI_) and initial decomposition rate × 100 (*k*_TBI_) from the teabag index. Also included is the vegetation coverage for graminoid (*%*_Gram._), forbs (*%*_Forbs_), evergreen shrubs (*%*_E‐shrub_), deciduous shrubs (*%*_D‐shrubs_), aerenchyma plants (*%*_Aeren._) for the five vegetation types.

	Treatment	Dry heath (HD)	Dry meadow (MD)	Mesic meadow (MM)	Wet meadow (MW)	Tussock tundra (TT)	References
SOM (2019)	*Ambient* *OTC*	0.2 ± 0.1 0.2 ± 0.0	0.5 ± 0.1 0.4 ± 0.1	0.5 ± 0.1 0.6 ± 0.1	0.4 ± 0.1 0.4 ± 0.1	0.1 ± 0.1 0.1 ± 0.0	This study
pH (2019)	*Ambient* *OTC*	5.0 ± 0.2 4.9 ± 0.1	6.6 ± 0.1 6.3 ± 0.1	6.4 ± 0.2 6.7 ± 0.1	6.2 ± 0.2 6.2 ± 0.2	5.3 ± 0.1 5.2 ± 0.1	This study
*C/N* (2008)	*Ambient* *OTC*	20.7 ± 0.3 23.6 ± 2.5	18.6 ± 1.0 20.1 ± 1.5	16.8 ± 1.0 17.7 ± 0.9	15.4 ± 0.4 15.4 ± 0.3	24.4 ± 1.6 24.8 ± 1.2	Scharn et al. (2022)
*S*_TBI_ (2016)	*Ambient* *OTC*	0.4 ± 0.0 0.4 ± 0.0	0.4 ± 0.0 0.3 ± 0.0	0.2 ± 0.1 0.2 ± 0.0	0.3 ± 0.0 0.3 ± 0.0	0.4 ± 0.0 0.4 ± 0.0	Sarneel et al. (2020)
*k*_TBI_ (2016)	*Ambient* *OTC*	1.3 ± 0.1 0.9 ± 0.0	1.3 ± 0.1 1.1 ± 0.1	1.5 ± 0.1 1.3 ± 0.1	1.2 ± 0.2 0.9 ± 0.3	0.9 ± 0.2 1.1 ± 0.2	Sarneel et al. (2020)
%_*Gram*._ (2016)	*Ambient* *OTC*	1.3 ± 0.8 0.3 ± 0.3	9.8 ± 2.6 14.0 ± 3.4	17.8 ± 4.2 10.6 ± 1.6	38.6 ± 4.3 23.6 ± 7.6	21.6 ± 3.5 25.2 ± 3.0	Scharn et al. (2022)
%_*Forbs*_ (2016)	*Ambient* *OTC*	0.3 ± 0.0 0.0 ± 0.0	18.0 ± 4.5 6.2 ± 2.9	8.8 ± 1.4 5.4 ± 1.9	27.6 ± 5.5 19.4 ± 5.1	1.4 ± 0.7 1.4 ± 0.9	Scharn et al. (2022)
%_*D‐shrub*_ (2016)	*Ambient* *OTC*	17.0 ± 4.9 19.8 ± 5.6	37.2 ± 2.8 7.0 ± 2.3	18.0 ± 5.1 25.0 ± 7.0	3.8 ± 2.2 20.2 ± 7.5	9.4 ± 2.5 7.8 ± 1.4	Scharn et al. (2022)
%_*E‐shrub*_ (2016)	*Ambient* *OTC*	15.5 ± 4.1 32.8 ± 5.2	14.6 ± 5.0 16.0 ± 5.5	12.0 ± 7.1 9.2 ± 3.3	0.0 ± 0.2 0.2 ± 0.2	16.4 ± 2.0 13.8 ± 3.5	Scharn et al. (2022)
%_*Aeren*._ (2016)	*Ambient* *OTC*	— —	5.0 ± 1.7 6.2 ± 2.3	3.8 ± 1.1 1.6 ± 0.4	4.0 ± 4.0 3.6 ± 2.0	21.4 ± 3.4 24.2 ± 2.4	Scharn et al. (2022)

*Note:* The year of measurement is indicated in brackets.

### 2.5 Statistical Approach

All statistical analyses were conducted in R version 4.0.0, Arbor Day (R Core Team 2020), unless otherwise stated. Missing *F*_net_ values for each measuring time point, vegetation type, and treatment were addressed by calculating the average of available data within each group, provided that only one or two (out of five) data points were missing. This approach facilitated the data structure required for the linear mixed‐effects model (LMM) analysis across all replicates.

To address non‐normality, positive and negative values, and high variances between different measurement time points, CH_4_ data were transformed and standardized using the *arcsinh* transformation in the R package *bestNormalize* (Peterson 2021) for each measurement time point and vegetation type separately. By this approach, a comparison of the treatment effect across a wide range of conditions while minimizing the seasonal variation is possible.

The effect size for each treatment within different communities was also calculated using Cohen's d, with Hedges' correction to account for the small sample sizes, using the *escalc* function of the R package *metafor* (Viechtbauer 2010). The effect size was calculated for each measurement time point.

The influence of the *OTC* treatment across communities and conditions was tested using a three‐step modelling approach. First, to assess significant differences in emissions and uptake from zero values for each vegetation type and treatment, a Wilcoxon signed‐rank test was used. Second, an LMM, using the *lme4* package (Bates et al. 2015), was used to test the influence of the *OTC* treatment across all communities while accounting for repeated measures at the same plot. Third, to disentangle the direct and indirect influence of the *OTC* treatment and *T*_soil_ and *SWC*, separate models were fit for each plant community, allowing for an interaction between treatment and *T*_soil_ and *SWC*. This approach was chosen to account for the hierarchical structure and high between‐groups variance of the data. Evaluation of effects was done using post hoc comparisons with *emmeans* (Lenth 2023) and Wald tests between contrasts.

LMMs were also used to analyse the influence of the *OTC* treatment and biological and environmental covariates (including ∑*T*_soil_, ∑*SWC*, *z*_canopy_, *%*_Gram._, *%*_Forbs_, *%*_E‐shrub_, *%*_D‐shrubs_, *%*_Aeren._ *%*_Erioph_, *k*_TBI_, and *S*_TBI_, were ∑ denotes the accumulated count for each year) on CH_4_ dynamics, with a random effect included to account for repeated measurements within the same communities. This was done using the full data set (LMM_full_) and for the *Ambient* and *OTC* treatment separately (LMM_*Ambient*_ and LMM_*OTC*_, respectively). The environmental covariates were tested for normality and collinearity, and CH_4_ data were transformed using a negative square root function to stabilize variance. Based on findings from the inhibition experiment (section 4), the TT community was excluded, and only data with *F*_net_ < 0 were used to specifically focus on net CH_4_ uptake driven by CH_4_ oxidation. This approach was chosen to minimize the complexities of concurrent methanogenesis and CH_4_ oxidation and improve model performance and interpretability. Separate models were used to check if *F*_net_ uptake is driven by different drivers in *Ambient* and *OTC* plots. Residuals were checked for normality and heteroscedasticity. While some violation of assumptions was indicated due to the complexity of the model process and the limited sample size, the models were considered valid to draw basic conclusions about the drivers of CH_4_ uptake.

For the wider survey, the *F*_net_, *F*_2022_ and *F*_Keuschnig_ were normalized and transformed using a signed log transformation. Data were then analyzed using LMM in the *lme4* package (Bates et al. 2015) with treatment and site as fixed effects, while replicates were included as a random intercept to account for repeated measures. Model fit was evaluated by comparing the full model against a random‐effects‐only null using likelihood ratio tests. Post hoc pairwise contrasts of estimated marginal means were conducted with the *emmeans* package (Lenth 2023), with Tukey adjustment for multiple comparisons.

The additional data on temperatures (*T*_*soil_TMS*_, *T*_*surface_TMS*_, and *T*_*air_TMS*_) and soil moisture (*SWC_*_*TMS*_) were analysed for the effects of treatment, vegetation type, and their interaction using a linear mixed‐effects model with year as a random effect. Growing season (JJA) averages of *T*_*soil_TMS*_, *T*_*surface_TMS*_, *T*_*air_TMS*_, and *SWC_*_*TMS*_ were analyzed during daylight conditions (9 am—5 pm), while the snowmelt was calculated from April onwards for each year.

In all analyses, the significance level was set to *p*‐value < 0.05 and, thus, referred to as significant in the following text. The detailed levels of significance will be given in tables and figures.

## 3 Results

### 3.1 CH_4_ Fluxes

**FIGURE 1:**
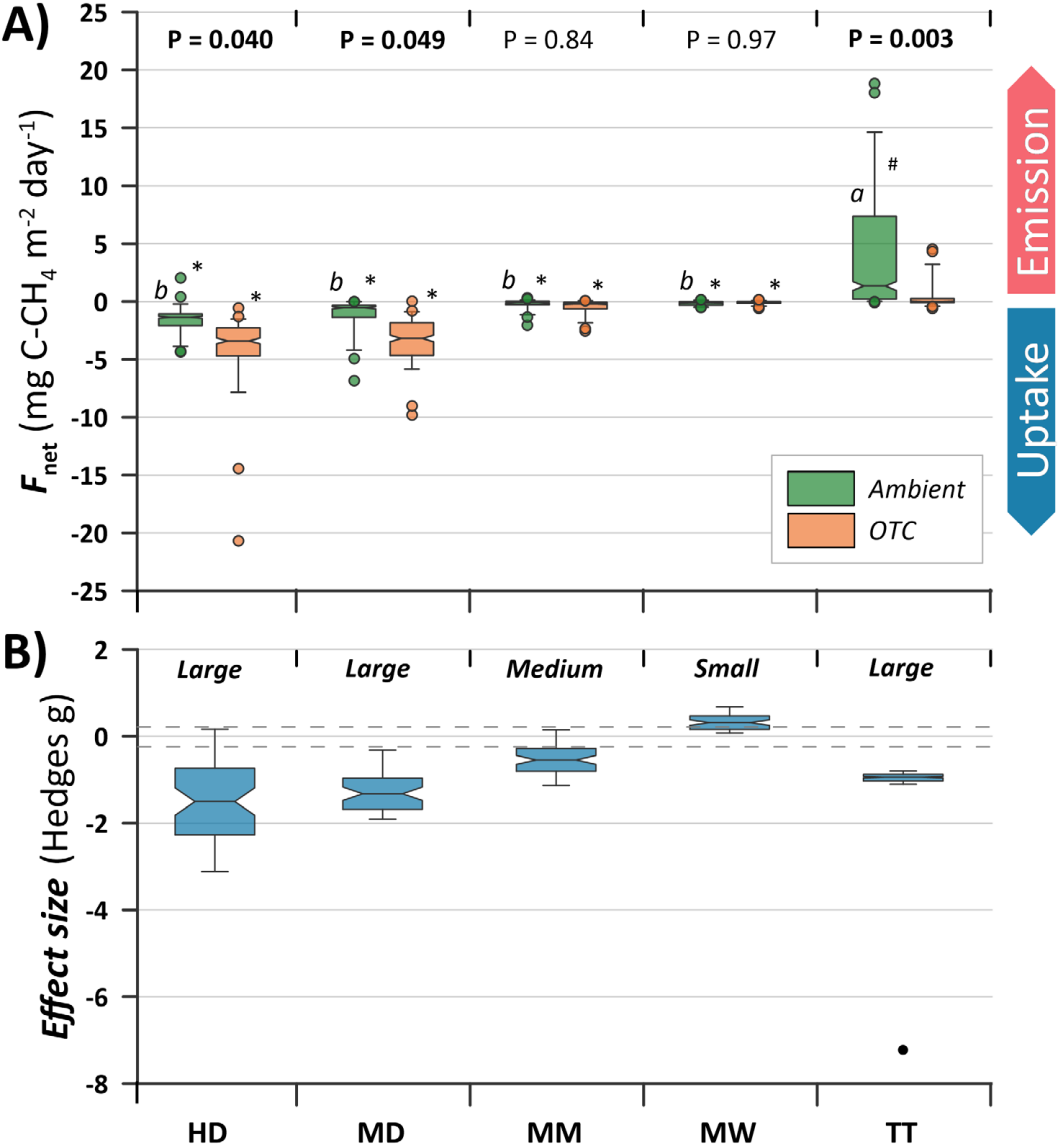
Summary of bi‐weekly growing season CH_4_ fluxes (2017 and 2018) in the five investigated plant communities, including: (A) Net CH_4_ fluxes (*F*_net_), (B) Hedges' g effect sizes determined from the normalized data. Notches indicate median, boxes start and end at the upper and lower quartiles, whiskers at the upper and lower 5 and 95 percentiles, and circles denote outliers. *p*‐values at the top of plot (A) indicate the difference between *Ambient* and *OTC* plots for each plant community. Different lower‐case letters indicate a significant difference among the *Ambient* plots in each plant community (*p* < 0.05). A “*” indicates a significant (*p* < 0.05) uptake, while a “#” indicates a significant (*p* < 0.05) production of CH_4_ when compared to a zero‐flux estimate.

The *Ambient F*_net_ varied between plant communities during the growing season of 2017 and 2018, with a significant net uptake in three of the plant communities: HD, MD, and MM (Figure 1A, Figure S2, Table S1). The *F*_net_ values were very small at the MW plots, showing low confidence of determination (i.e., no measurements with *R* ^2^ > 0.8). The TT was the only plant community with a consistent net CH_4_ emission (*F*_net_ > 0) in the *Ambient* plots (Figure 1A, Figure S2). The *OTC* treatment significantly increased the CH_4_ uptake rates (*F*_net_ < 0) in two communities, with an overall 140.4% ± 8.1% and 204.2% ± 19.3% increase in HD and MD, respectively, while no significant difference was found in the MM and MW communities (Figure 1A, Figure S2). At the TT site, the *OTC* treatment significantly reduced the emissions rates by 91.2% ± 18.6%, occasionally causing the net emission to become a net uptake (Figure 1A, Figure S2).

Neither *Ambient* nor *OTC* plots differed in *F*_net_ between years (2017 and 2018) in any of the plant communities, except for the *OTC* plots in HD, which showed a higher CH_4_ uptake in 2018 (Table S1). Additionally, July mean daily air temperature differed significantly between 2017 (7.3°C ± 3.8°C) and 2018 (12.4°C ± 4.1°C) (LFS monitoring, data not shown). In comparison to *F*_2022_ (TT and MD) and *F*_Keuschnig_ (TT), the *F*_net_ from the *OTC* treatment had significantly increased CH_4_ uptake rates in MD and reduced emissions in TT (Figure S3). The *Ambient F*_net_, however, did not differ from *F*_2022_ nor *F*_Keuschnig_ (Figure S3).

### 3.2 CH_4_ Normalized Data and Effect Sizes

By normalizing the *F*_net_ for both *Ambient* and *OTC* plots for each plant community and measuring occasion, the overall treatment effect size was calculated, in which we removed the effect of time and seasonal environmental conditions (e.g., temperature, soil moisture and plant phenological stages), thereby indicating changes in oxidation capacity. An increased oxidation capacity is here defined as either increased uptake, reduced emission, or a combination of both. This approach showed an increase in the oxidation capacity in four of the plant communities, were HD, MD, MM and TT show a medium to large negative effect size, while MW showed a small positive effect size (indicating increased emissions with *OTC* treatment) (Figure 1B). No seasonal shifts were found in the effect sizes, except for MM in 2018 and TT in 2017. At those occasions, the shifts indicated increased oxidation with seasonal progression (Figure S4).

### 3.3 CH_4_ Inhibition

**FIGURE 2:**
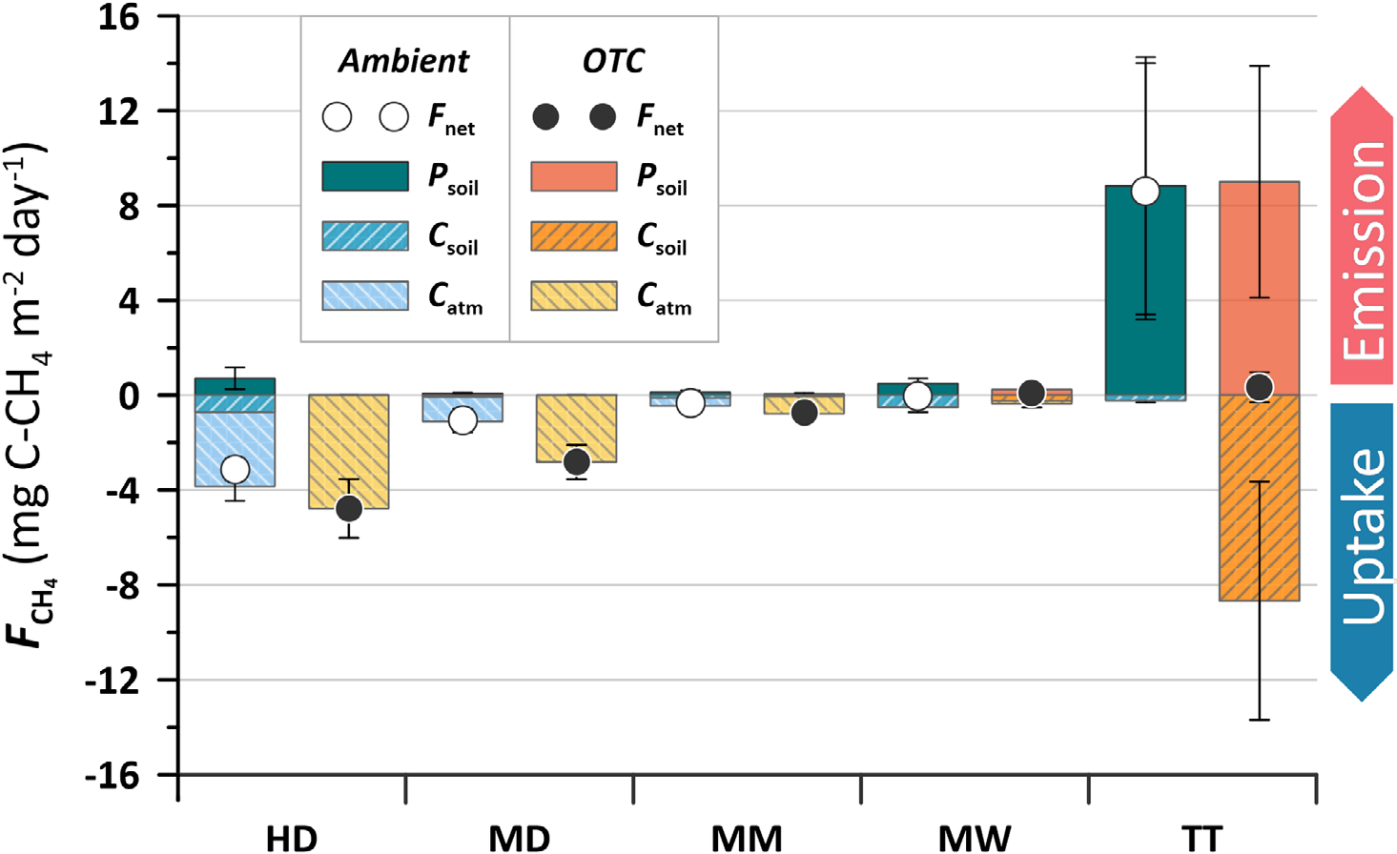
The estimated relationship between net fluxes (*F*_net_), soil production (*P*_soil_) and consumption of atmospheric (*C*_atm_) as well as soil‐produced (*C*_soil_) CH_4_ as revealed during the inhibition experiment in 2019.

The inhibition experiment in 2019 revealed *P*_soil_ in all plant communities (Figure 2), with *P*_soil_ detectable in two to five of the *Ambient* plots per plant community. The largest production of CH_4_ occurred at the TT *Ambient* plots. *OTC* plots had low or non‐detectable *P*_soil_, at HD, MD, MM, and MW. However, TT showed a similar *P*_soil_ in both *OTC* and *Ambient* plots when the CH_4_ oxidation was inhibited (Figure 2), contrasting the *F*_net_ fluxes before inhibition (Figure 1A). The total oxidation (*C*_soil_ + *C*_atm_) was significant from zero in all plant communities and treatment, except for the *OTC* plots in TT being marginally significant (*p* = 0.08).

### 3.4 Plant Community and Edaphic Factors

**FIGURE 3:**
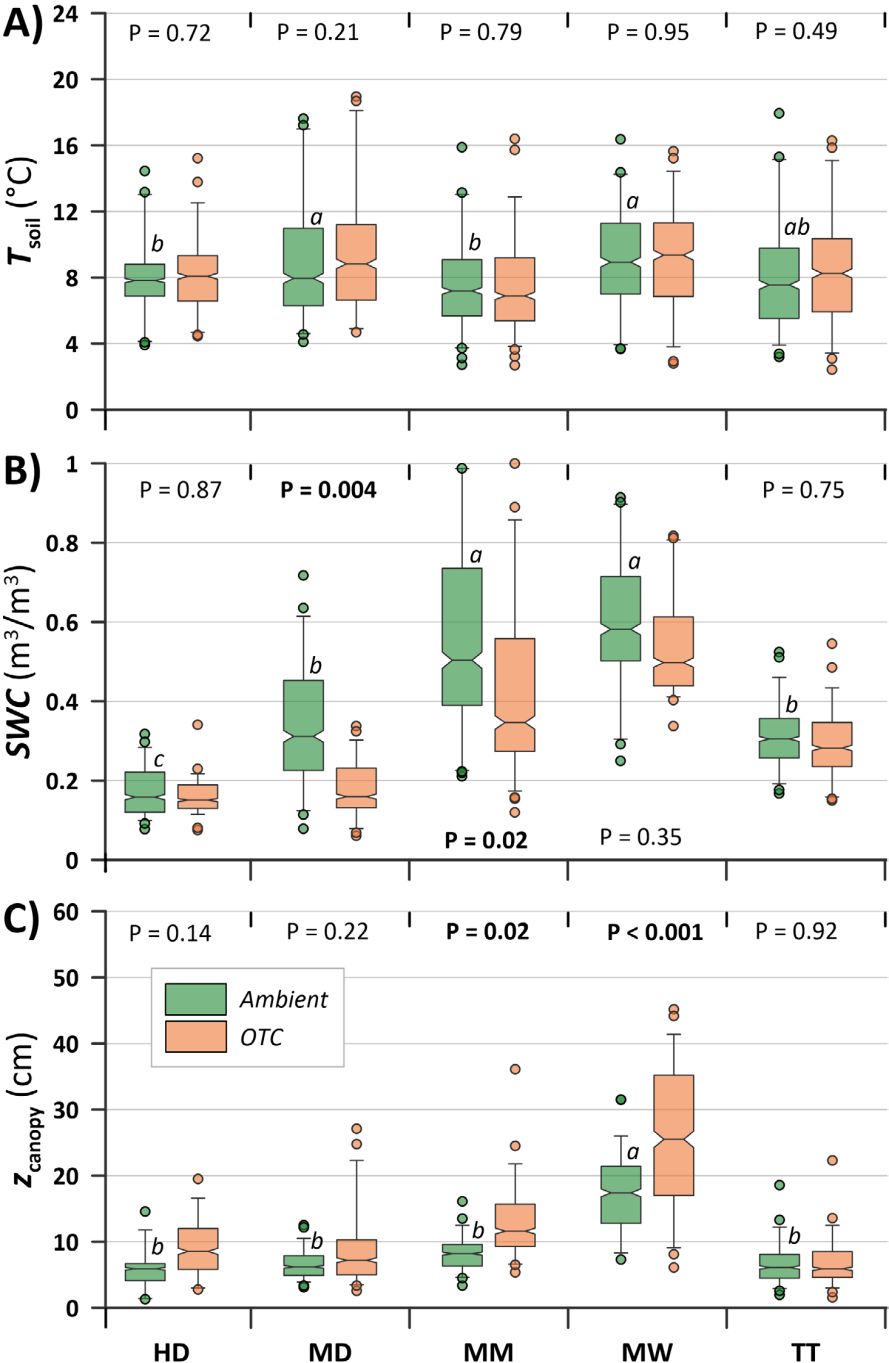
Environmental parameters, including bi‐weekly (2017 and 2018) measurements of: (A) soil temperature data (*T*_soil_) at −5 cm and (B) soil water content (*SWC*) at 0–6 cm, as well as (C) canopy height (*z*_canopy_) measured in 2020. Notches indicate median, boxes start and end at the upper and lower quartiles, whiskers at the upper and lower 5 and 95 percentiles, and circles denote outliers. Stated *p*‐values indicate a difference between *Ambient* and *OTC* plots. Different lower‐case letters indicate a significant difference among the *Ambient* plots in each plant community (*p* < 0.05).

The *T*_soil_, measured along each *F*_net_ measurement, was found unaffected by the *OTC* treatment (Figure 3A) and was similar between plant communities. In contrast, the *SWC* differed significantly between plant communities, with the MM and MW being the wettest, and HD the driest (Figure 3B). Significant drier conditions were found in the *OTC* plots of MD and MM (Figure 3B). No treatment difference in *SWC* was found in the HD, MW, and TT communities (Figure 3B), and only the MD community had significant differences between the year (data not shown). Furthermore, the daytime temperatures of *T*_*air_TMS*_ and *T*_*surface_TMS*_ showed significant increases in all plant communities with OTC treatment (Table S2), averaging 2.7°C ± 0.1°C and 2.0°C ± 0.1°C, respectively, while including nighttime temperatures dampened the effect to 1.1°C ± 0.1°C and 1.0°C ± 0.1°C, respectively (data not shown). For *T*_*soil_TMS*_, only MD stood out as significant, with a moderate increase of 0.4°C ± 0.1°C. The *SWC_*_*TMS*_ indicates significant dryer conditions with *OTC* treatment in all plant communities except TT, which was slightly wetter (Table S2). The *DSM_*_*TMS*_ reveals a three to 7‐day earlier melt‐out in the *OTC* plots compared to the controls in HD, MD, MM, and MW, while the *OTC* plots in TT melted out with a 2‐day delay (Table S2).

The vegetation heights measured in 2019 revealed similar *z*_canopy_ in the *Ambient* plots in all plant communities apart from MW, which had significantly 2.5 times higher vegetation than the other communities (Figure 3C). The *OTC* treatment showed significantly higher *z*_canopy_ in the MM and MW plant communities while indicating an increasing trend (*p*‐values between 0.14 and 0.22) in HD and MD (54.1% ± 15.5%, 35.0% ± 19.9%, 61.1% ± 8.5%, and 50.1% ± 9.8% for HD, MD, MM, and MW, respectively). TT showed no difference between *Ambient* and *OTC* plots (Figure 3C). The calculated *m*_Bio_ of the field layer in the *Ambient* plots was similar for all plant communities except MW, which was significantly 1.6 times higher than the other communities. The *OTC* treatment generated highly varying responses in the calculated *m*_Bio_, except for MD and MW, with a significant increase by 163.8% ± 116.0% and 97.3% ± 240.0%, respectively (Figure S5).

### 3.5 Q_10_ and SWC Relationships

Significant Q_10_ estimates for the *F*_net_ uptake rates (*F*_net_ < 0) were only found among parts of the plant communities and treatments, including the *Ambient* plots at the MD and MM and the *OTC* plots of the HD, MM and MW (Figure S6). The *SWC* indicates a large range of soil water regimes (0.06 to 1.00 m^3^/m^3^) when including all plant communities, where the corresponding *F*_net_ shows both the highest uptake and emission rates at *SWC* below 0.52 m^3^/m^3^ (Figure S7). Furthermore, a second‐order polynomial function was fitted (Table 1), according to D'Imperio et al. (2017), between *F*_net_ uptake rates and *SWC*, including all plant communities, indicating a higher CH_4_ uptake rate at lower SWC (< 0.4 m^3^ m^−3^) and pronounced in the *OTC* plots (Figure S7F).

### 3.6 CH_4_ Fluxes—Explanatory Factors

The LMM, including the treatment effect as well as *T*_soil_ and *SWC* directly linked to each *F*_net_ measurement, revealed deviating explanatory factors for the individual plant communities (Table 2). Note, when constructing LMMs for data including both uptake rates and emissions of CH_4_ (indicated by negative or positive *F*_net_, respectively), the generated positive or negative direction of the LMM includes both processes. A negative trend then indicates an increased uptake of CH_4_ or a reduced emission of the same, and vice versa for positive values. At HD, the treatment resulted in a significant increase in observed CH_4_ uptake, while no effect was observed for *T*_soil_ and *SWC*. The MD had a similar effect of the treatment and, in addition, a significant effect of *T*_soil_, with increasing CH_4_ uptake when *T*_soil_ increases. The MM showed no direct influence of the treatment, but increased *SWC* was associated with decreased CH_4_ uptake rates (or increased emissions of the same). At the MW, increasing *T*_soil_ showed increases in CH_4_ uptake, while increasing *SWC* was linked to reduced CH_4_ uptake rates (or increased emissions of the same), with no effect of the treatment itself. At the TT, the treatment was associated with declining emission rates (or increased uptake rates of the same) while an increase in *T*_soil_ was associated with enhanced CH_4_ emissions (or reduced CH_4_ uptake rates).

**TABLE 2:** Summary of explanatory factors and their significance based on the LMM for each plant community, using all paired *F*_net_, *T*_soil_ and *SWC* measurements for both *Ambient* and *OTC* plots to reveal the treatment effect.

	Treatment	*T*_soil_	*SWC*
HD	(−)*		
MD	(−)*	(−)*	
MM			(+)***
MW		(−)**	(+)***
TT	(−)**	(+)*	

*Note:* Stars indicate significance level: ***p < 0.001, **p < 0.01, *p < 0.05 of each factor. Negative signs indicate increased CH_4_ uptake rates (or a reduction of emissions), while positive signs indicate decreased CH_4_ uptake rates (or increased emissions).

The LMM_full_, investigating the full set of environmental parameters (Table 3), revealed an overall strong treatment effect, with increased CH_4_ uptake rates (or decreased emissions) with *OTC* treatment. An increase in *SWC* was found to decrease the CH_4_ uptake (or increase emissions) in LMM_full_, as well as in the LMM_*OTC*_ (using only the treated plots), while *T*_soil_ only showed an influence for the LMM_*OTC*_ where higher *T*_soil_ resulted in increased CH_4_ uptake rates (or decreased emissions). The LMM_full_ further reveals an increased CH_4_ uptake (or decreased emissions) with higher C/N and %_E‐shrub_, while increasing *z*_canopy_, *%*_Gram_, and *%*_Forbs_ reduce the CH_4_ uptake (or increased emissions). In LMM_*Ambient*_, only the *%*_Gram_ showed a significant relation, with an increased *%*_Gram_ reducing CH_4_ uptake rates (or increasing emissions). LMM_*OTC*_ revealed reduced CH_4_ uptake rates (or increased emissions) with increases in *S*_TBI_, *k*_TBI_, *%*_Gram._, *%*_Forbs_, *%*_D‐shrubs_, whereas increasing *T*_soil_ and C/N ratios lead to increased CH_4_ uptake (or reduced emissions).

**TABLE 3:** Summary of explanatory factors from the LMMs combining all plant communities, using seasonally cumulative numbers (∑) for *T*_soil_ and *SWE*.

	Treat	∑*T*_soil_	∑*SWC*	SOM	pH	C/N	*S*_TBI_	*k*_TBI_	%_*Gram*._	%_*Forbs*_	%_*D‐shrub*_	%_*E‐shrub*_	%_*Aeren*._	z_canopy_
LMM_full_	(−)**		(+)*		(+)**	(−)*	(+)***		(+)**	(+)***		(−)**		(+)***
LMM_*Ambient*_									(+)***					
LMM_*OTC*_		(−)*	(+)*			(−)***	(+)***	(+)***	(+)**	(+)***	(+)**			

*Note:* Stars indicate significance level: ***p < 0.001, **p < 0.01, *p < 0.05, where factors missing stars did not influence the final model. Negative signs indicate increased CH_4_ uptake rates (or a reduction of emissions), while positive signs indicate decreased CH_4_ uptake rates (or increased emissions).

## 4 Discussion

Here, we demonstrate that Arctic ecosystems, after 23–25 years of passive air warming treatment, can increase the total soil CH_4_ oxidation capacity. This is due to both enhanced uptake of atmospheric CH_4_, as well as oxidizing CH_4_ produced within the soil, reducing net emission rates from communities with CH_4_ production, corroborating earlier results from both wet and dry Arctic soils (D'Imperio et al. 2017; Pedersen et al. 2018; Zheng et al. 2018). Importantly, our observed treatment‐induced increase in CH_4_ uptake is substantially higher than previously indicated from a Low‐arctic tundra in Western Greenland (33% increase in a dry heath community) (D'Imperio et al. 2017). Thus, our results give strong evidence of the importance of including CH_4_ oxidation processes in all ecosystem types in future regional CH_4_ assessments.

Methanotrophic inhibition revealed CH_4_ production in all investigated plant communities, underscoring the importance of methanogenic processes even in very dry soils (HD and MD). This highlights that the CH_4_ uptake observed in *F*_net_ represents only a fraction of the total CH_4_ oxidation capacity occurring within the soil. It confirms that low and zero *F*_net_ should not be excluded when analysing CH_4_ fluxes from Arctic sites. The production of CH_4_ observed in our dry communities, such as the *Ambient* plots of HD and MD, is likely due to microsites with low redox potential within the soil (Megonigal and Guenther 2008), where both the HD and MD communities are situated on moderate slopes with no standing water nearby. Surprisingly, no *P*_soil_ was observed in the *OTC* plots at HD and MD, indicating that the long‐term air warming treatment affects these low‐redox microsites. The small *P*_*soil*_ observed within the *Ambient* HD and MD plots likely provides sufficient additional resources to support a slightly larger methanotrophic community, increasing *C*_atm_, but further microbial assessment is needed to confirm the status of the CH_4_‐cycling community. Furthermore, the diffusion rate and efficiency of the acetylene gas might differ between different soils and water contents (Ryden and Dawson 1982), which might imply some uncertainties in the results. However, the method has little effect on *P*_soil_ (Ding et al. 2004; King 1996), indicating that if the inhibition here is incomplete, then the *P*_soil_ is likely underestimated and so are the consumption rates.

The MW community investigated here, surprisingly, shows an almost complete absence of CH_4_ activity, close to zero fluxes, in both the bi‐weekly flux measurements and during the inhibition experiment. Earlier studies (Mohanty et al. 2006; Zhuang et al. 2013) have indicated that the presence of ammonium (NH_4_) in the soil can inhibit the oxidation capacity of methanotrophs since the *pmoA* enzyme (methane monooxygenase), carrying out the first step in CH_4_ oxidation, also has an affinity for NH_4_. Similar effects can also be observed at lower pH (Le Mer and Roger 2001). However, the NH_4_ levels in the MW are in the same range as for MD and MM and comparably low compared to TT and HD, and pH is in the midrange of all communities (Scharn et al. 2022), indicating other unfavourable conditions for CH_4_‐cycling may apply. The TT community displayed a high *P*_soil_ in both *Ambient* and *OTC* plots, while net CH_4_ emissions were only pronounced under ambient conditions. Interestingly, the calculated mean *C*_soil_ in the *OTC* plots at TT did not match the calculated means in the *Ambient* plots despite similar *SWC* and *T*_soil_. The presence of aerenchyma vegetation can influence both *C*_soil_ and CH_4_ emission rates by gas diffusion through its tissue (Chanton et al. 1992; Ström et al. 2012). Although a clear decrease in *E. vaginatum* has been observed in the TT community over the monitoring period, due to the loss of underlying permafrost (Beylich et al. 2004, 2003; Keuschnig et al. 2022; Molau 2010), there is no difference in the *E. vaginatum* cover between the *Ambient* and the *OTC* plots (Scharn et al. 2022). Instead, we consider the shift from a sedge‐dominated plant community towards a heath tundra ecosystem, more pronounced under long‐term air warming (Scharn et al. 2022), has influenced the net CH_4_ budget by increasing aeration of the microenvironment in the top few centimetres of the soil. A similar pattern was observed when this TT community was compared to a tussock tundra community at Corrvosjávri (12 km south and 200 m lower in elevation), a site with a longer post‐permafrost period and with an advanced stage of shrubification (Keuschnig et al. 2022). The CH_4_ emission levels observed in the *OTC* plots at the TT (0.35 ± 0.15 mg C‐CH_4_ m^−2^ day^−1^) are very similar to what has been observed at the Corrvosjávri tussock tundra (0.35 ± 0.12 mg C‐CH_4_ m^−2^ day^−1^) (Keuschnig et al. 2022). Furthermore, among the shrubs advancing in our TT, MM and MD communities, the evergreen boreal dwarf‐shrub *V. vitis‐idaea* is especially favoured with a higher abundance under long‐term air warming (Molau 2010; Scharn et al. 2022). Interestingly, the presence of *V. vitis‐idaea* plant roots has been found to enhance methanotrophy (Halmeenmäki et al. 2017), thereby increasing CH_4_ uptake in the soil. These findings suggest that the shift in plant community composition, particularly the increase in *V. vitis‐idaea* , plays a crucial role in enhancing CH_4_ uptake and altering CH_4_ dynamics.

We observed no treatment effect on *T*_soil_ during the investigated period. This aligns with previous findings from our study site (Scharn et al. 2021) but deviates from the overall pattern of this experimental design, which typically results in a slight increase in soil temperatures (approx. 0.4°C) (Maes et al. 2024). The OTC treatment effect on *T*_soil_ and *SWC* tends to vary between sites, soil moisture, plant communities and experiment duration (Bokhorst et al. 2013; Hollister et al. 2023). This variability is likely due to a combination of factors, including the small plot size (1 m^2^), lateral water flow (Scharn et al. 2021), and the shading effect of increased vegetation (Hollister et al. 2023). The moist to wet plant communities at our study site are influenced by lateral water movements (MM and MW) or higher water table (TT), slowly drying up over the season when the uphill snow beds diminish and soils thaw, increasing the drainage (Molau 2010; Scharn et al. 2022, 2021). The shading effect likely also plays a role here, where taller vegetation (MM and MW) and increased bottom layer coverage (TT and HD) had been observed both in this and earlier studies (Baruah et al. 2017; Molau 2010; Scharn et al. 2022). Furthermore, the treatment influence on *SWC* has earlier been attributed to air temperature‐mediated drying and has been found to be an important driver of diversity and vegetation transition among these plant communities (Scharn et al. 2021). The increase in near‐surface air temperature generated by the OTCs (Marion et al. 1997), along with the increased width and length of leaves (Baruah et al. 2017), likely enhances evapotranspiration rates, leading to the drying of surface soils, as observed in similar experiments in Greenland and Canada (Dabros et al. 2010; D'Imperio et al. 2017). Total biomass also likely contributes to this effect, but our calculated *m*_bio_ deviates from the expected general increase (Pearson et al. 2013), especially for the MD community. The deviation in MD is due to a snow mould‐initiated die‐back of *D. octopetala* during 2015 and 2016 and a lack of bottom‐layer biomass data. These findings underscore the complexity of ecological responses to experimental treatments and highlight the importance of accounting for ecological events to consider site‐specific conditions and dynamics.

Our LMM modelling did not give a uniform response to soil temperature as the main predictor for CH_4_ oxidation among the plant communities, mainly due to a lack of soil temperature difference between treatments. This suggests that other processes, such as the increased evapotranspiration‐induced soil drying, might be of importance. For the specific plant communities investigated here, the timing of snow melt and the duration of long‐lasting snowpacks uphill from the plant communities directly influence soil moisture conditions (Scharn et al. 2021). The year‐round deployment of OTCs at our site likely has a limited effect on snow conditions (height and durations), only moderately changing the conditions, leaving the landscape distribution of snow a larger predictor of soil moisture, and thus influencing the CH_4_ oxidation capacity. Given the general Arctic trend of increasing growing season length, and with some Arctic regions showing a reduction in snow cover (Arndt et al. 2019; Blinova and Chmielewski 2015; Derksen and Brown 2012; Høgda et al. 2013; Karlsen et al. 2022), soil drying is expected to co‐occur, as indicated by recent modelling efforts (Andresen et al. 2020). Additionally, an increase in shrub abundance (Elmendorf, Henry, Hollister, et al. 2012; Keuschnig et al. 2022; Myers‐Smith et al. 2011), as visible in some but not all of our plant communities, might enhance soil aeration. These factors together could facilitate increased CH_4_ oxidation rates at larger regional scales. Our results point towards even higher potential future Arctic CH_4_ oxidation rates than the pure temperature dependence pointed out by Oh et al. (2016, 2020), further mitigating the large CH_4_ emissions estimated from the region.

## 5 Conclusion

**FIGURE 4:**
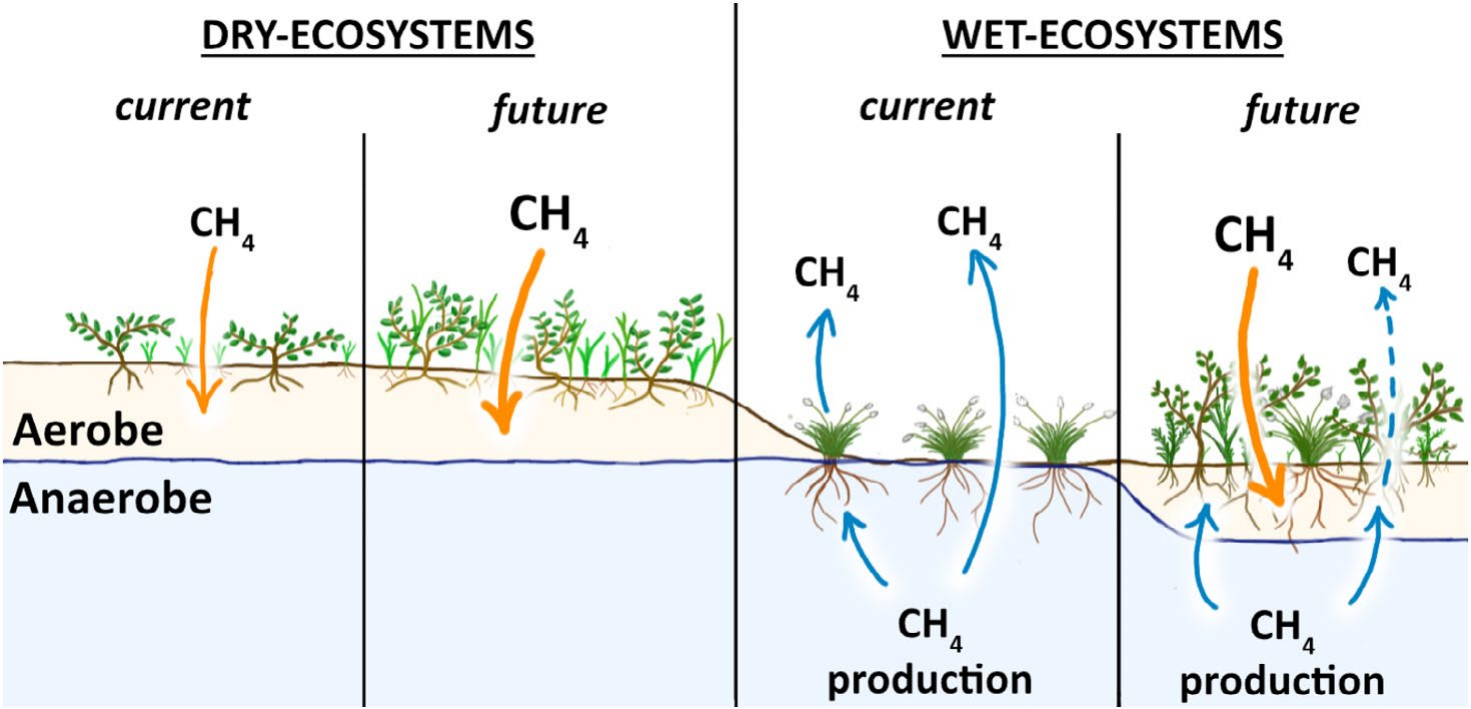
Conceptual framework, where a future climate, with changed plant communities (including taller plants, increased biomass, and changed plant communities), initiates an increase in vegetation‐mediated evapotranspiration and, thus, favours the uptake of atmospheric CH_4_ and reduces CH_4_ emissions.

The warming‐induced changes in vegetation and soil moisture regimes across the five plant communities at Latnjajaure Field Station provide compelling evidence for increased soil CH_4_ oxidation capacity. This increase in oxidation capacity includes increased uptake rates of atmospheric CH_4_ (high‐affinity methane oxidation), pronounced in drier soils, as well as reduced net CH_4_ emission (low‐affinity methane oxidation) from methanogenic processes occurring under anaerobic conditions in deeper soil layers. While increasing air temperatures have been suggested to enhance the methanotrophic oxidation rates of CH_4_ in the Arctic, for example, Oh et al. (2020), our results show that vegetation‐mediated soil drying and shifts in vegetation composition are equally critical drivers of CH_4_ oxidation in both dry and wet Arctic ecosystems (Figure 4). These vegetation‐soil interactions imply that future CH_4_ oxidation rates may be higher than recent model predictions (Oh et al. 2020, 2016) once vegetation feedbacks are considered, thereby mitigating regional net emission scenarios. Together with prior studies in Arctic ecosystems with low soil organic content (D'Imperio et al. 2023; Juncher Jørgensen et al. 2024, 2015; Stackhouse et al. 2017), our findings argue for a more holistic perspective of CH_4_ dynamics, integrating above and below‐ground processes and their interactions, across both future climate predictions and in situ investigations. By elucidating how vegetation change and soil moisture regulate CH_4_ oxidation, this study helps to constrain a previously poorly understood component of the global CH_4_ budget, thereby improving the basis for projecting atmospheric CH_4_ trajectories and associated radiative forcing. This advances the field from recognizing CH_4_'s growing atmospheric impact to quantifying a key terrestrial sink mechanism that will shape Arctic—and global—CH_4_ outcomes under continued warming.

## Author Contributions

**Mats P. Björkman:** conceptualization, data curation, formal analysis, funding acquisition, investigation, methodology, project administration, resources, supervision, validation, visualization, writing – original draft, writing – review and editing. **Jan Dietrich:** data curation, formal analysis, validation, writing – original draft, writing – review and editing. **Mabel L. Gray:** investigation, writing – review and editing. **Argus Pesqueda:** investigation, writing – review and editing. **Mario Rudner:** investigation, writing – review and editing. **Laura Rasmussen:** formal analysis, writing – review and editing. **Joel D. White:** investigation, writing – review and editing. **Bo Elberling:** conceptualization, writing – review and editing. **Robert G. Björk:** conceptualization, writing – review and editing.

## Funding

This work was supported by the H2020 Marie Skłodowska‐Curie Actions (657627 to M.P.B.); Svenska Forskningsrådet Formas (2016‐01187 and 2022‐00786 to M.P.B.); the Swedish Research Council (2021‐04011 to M.P.B. and 2023‐04048 to R.G.B.); Danmarks Grundforskningsfond (CENPERM DNRF100 to B.E.); and Danmarks Frie Forskningsfond (1059‐00003B to L.H.R.). Additional funding was provided by BECC (Biodiversity and Ecosystem Services in a Changing Climate) and the foundations of H. Ax:son Johnson, Carl Tryggers, Knut & Alice Wallenberg, and Wilhelm & Martina Lundgren to M.P.B.

## Conflicts of Interest

The authors declare no conflicts of interest.

## Supporting information


**Data S1:** gcb70810‐sup‐0001‐Supinfo.pdf.

## Data Availability

The data that support the findings of this study are openly available through the Swedish National Data Service: https://doi.org/10.5878/y091‐aq58.
