# Supplementary material for: Increased CH4 Oxidation in Arctic Tundra Ecosystems Caused by Vegetation‐Mediated Soil Drying
Source: Glob Chang Biol. 2026 Mar 26;32(3):e70810. doi: 10.1111/gcb.70810 (PMC13019277; doi:10.1111/gcb.70810)
Supplement: Supplementary file 1 — Data S1: gcb70810‐sup‐0001‐Supinfo.pdf. [file GCB-32-e70810-s001.docx]

*Supplement material:*

Title:  **Increased CH_4_ oxidation in Arctic tundra ecosystems caused by vegetation-mediated soil drying.**

Authors: Mats P. Björkman^1, 2^*, Jan Dietrich^3^, Mabel L. Gray^4^, Argus Pesqueda^5^, Mario Rudner^6^, Laura Rasmussen^7, 8^, Joel D. White^1^, Bo Elberling^9^, Robert G. Björk^2, 6^

Affiliations: ^1^Department of Biological and Environmental Sciences, University of Gothenburg, Box 463, SE-40530 Gothenburg, Sweden.

^2^Gothenburg Global Biodiversity Centre, Box 461, SE-405 30 Gothenburg, Sweden.

^3^Department of Ecology and Environmental Science, University of Umeå, SE-901 87 Umeå, Sweden.

^4^Department of Earth and Planetary Sciences, American Museum of Natural

History, New York, NY 10024, U.S.

^5^Center for Ecological Research and Forestry Applications, Edifici C, Universitat Autònoma de Barcelona, 08193 Bellaterra, Barcelona, Spain

^6^Department of Earth Sciences, University of Gothenburg, Box 460, SE-40530 Gothenburg, Sweden.

^7^Copenhagen Data Lab, Department of Mathematical Sciences, University of Copenhagen, Denmark.

^8^Department of Geoscience, University of Tromsø, Tromsø, Norway.

^9^Department of Geosciences and Natural Resource Management, Øster Voldgade 10, DK-1350 Copenhagen K., Denmark.

*Corresponding author: Mats P. Björkman

Department of Biological and Environmental Sciences, University of Gothenburg, Box 463, SE-40530 Gothenburg, Sweden

E-mail: mats.bjorkman@bioenv.gu.se

Telephone number: +46 766 18 18 20

**Table S1**. Summary of CH_4_ fluxes and Hedges' g effect sizes (x̅ ± S.E.) for each plant community over the measurement period.

| **Parameter** | **Treatment** | **Period** | **HD** | **MD** | **MM** | **MW** | **TT** |
| --- | --- | --- | --- | --- | --- | --- | --- |
| mg C-CH_4_ m^-2^ day^-1^ | *Ambient* | 2017 | -1.79±0.15 | -1.40±0.41 | -0.20±0.08 | -0.13±0.04 | 4.28±1.14 |
| mg C-CH_4_ m^-2^ day^-1^ | *OTC* | 2017 | -2.75±0.19 | -3.33±0.36 | -0.34±0.07 | -0.06±0.02 | 0.55±0.30 |
| mg C-CH_4_ m^-2^ day^-1^ | *Ambient* | 2018 | -1.65±0.27 | -0.86±0.19 | -0.21±0.07 | -0.16±0.04 | 3.76±0.99 |
| mg C-CH_4_ m^-2^ day^-1^ | *OTC* | 2018 | -6.10±0.93 | -3.35±0.51 | -0.58±0.15 | -0.11±0.03 | 0.17±0.11 |
| mg C-CH_4_ m^-2^ day^-1^ | *Ambient* | 2017+2018 | -1.75±0.14 | -1.10±0.21 | -0.21±0.05 | -0.15±0.03 | 4.01±0.74 |
| mg C-CH_4_ m^-2^ day^-1^ | *OTC* | 2017+2018 | -4.17±0.46 | -3.34±0.32 | -0.45±0.08 | -0.09±0.02 | 0.35±0.15 |
| Hedges' g |  | 2017 | -0.88±0.34 | -1.27±0.34 | -0.49±0.18 | 0.45±0.10 | -0.87±0.03* |
| Hedges' g |  | 2018 | -2.44±0.33 | -1.37±0.14 | -0.61±0.16 | 0.23±0.08 | -1.02±0.03 |
| Hedges' g |  | 2017+2018 | -1.50±0.34 | -1.32±0.16 | -0.54±0.12 | 0.32±0.07 | -0.95±0.03* |
| *An * indicates values where the outlier from the first measurements of the 2017 season has been excluded. See Fig. 2 in the main paper and Fig. S2 for more details.* | | | | | | | |

**Table S2**. Summary of soil temperature at – 8 cm (*T*_soil_TMS_), surface temperature (*T*_surface_TMS_) and air temperature at 15 cm (*T*_air_TMS_) data, as well as soil moisture data (*SWC_*_TMS_) and day of snow melt (*DSM_*_TMS_) as day of year (DOY), all with standard deviation. Average temperatures and moisture were analyzed during June, July and August between the hours of 9 am – 5 pm. While the snowmelt was calculated the first day of three consecutive days without snow after 1^st^ of April each year.

| **Parameter** | **Treatment** | **Unit** | **HD** | **MD** | **MM** | **MW** | **TT** |
| --- | --- | --- | --- | --- | --- | --- | --- |
| *T_air_TMS_* | *Ambient* | °C | 11.9±5.5 | 12.3±5.3 | 12.4±5.4 | 12.4±5.4 | 11.8±5.4 |
| *T_air_TMS_* | *OTC* | °C | 14.6±6.0* | 14.82±5.9* | 14.8±5.6* | 15.1±5.8* | 15.0±6.4* |
| *T_surface_TMS_* | *Ambient* | °C | 12.3±5.6 | 12.77±5.0 | 12.6±5.1 | 11.94.9± | 12.3±5.6 |
| *T_surface_TMS_* | *OTC* | °C | 14.5±6.1* | 14.3±5.4* | 14.1±5.1* | 13.6±5.1* | 15.3±6.6* |
| *T_soil_TMS_* | *Ambient* | °C | 8.7±4.3 | 9.2±3.7 | 7.7±3.8 | 8.1±4.2 | 8.5±4.4 |
| *T_soil_TMS_* | *OTC* | °C | 8.4±4.1 | 9.6±3.7* | 7.8±3.9 | 8.3±4.4 | 8.3±4.4 |
| *SWC__TMS_* | *Ambient* | % | 14.2±5.3 | 26.5±9.2 | 26.4±1.1 | 36.7±1.5 | 22.0±6.9 |
| *SWC__TMS_* | *OTC* | % | 10.0±6.3* | 16.3±6.5* | 19.0±9.4* | 32.5±1.4* | 23.4±8.8* |
| *DSM__TMS_* | *Ambient* | DOY | 143±7 | 133±4 | 130±7 | 146±3 | 142±8 |
| *DSM__TMS_* | *OTC* | DOY | 146±5 | NA | 125±13 | 138±5 | 144±7 |

*A * indicates a significant (p < 0.05) difference from the Ambient plots. The data for DSM__TMS_ did not allow for statistical analysis due to missing values.*


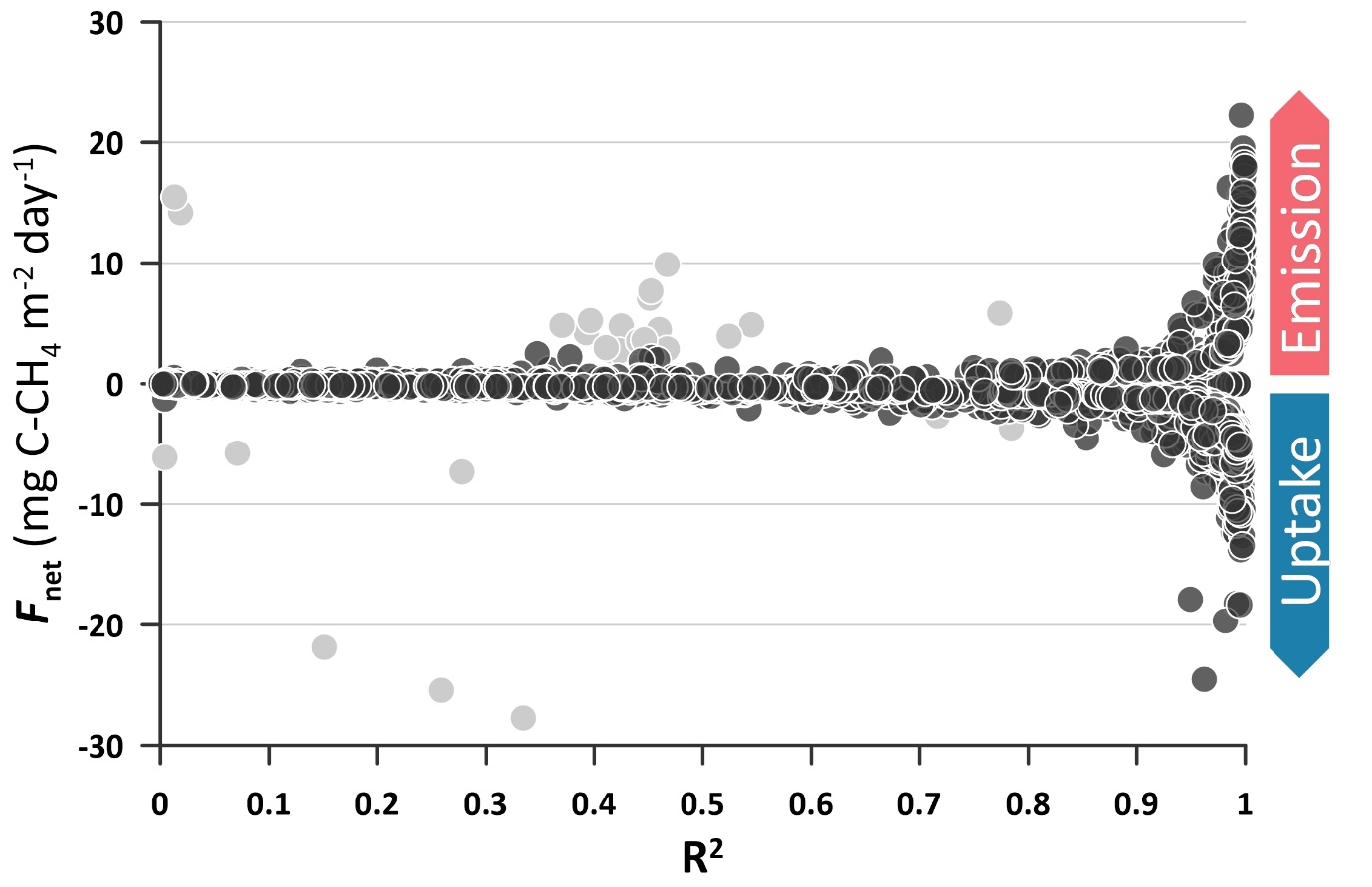


**Figure S1**. Measured net fluxes of CH_4_, *F*_net_, as a function of the obtained coefficient of determination, R^2^, of the linear regression established when calculating the flux. Data includes all measurements from the 2017 and 2018 campaigns (n = 4,117), with grey-marked indicating excluded outliers (3 x σ) for the range 0 < R^2^ < 0.8 (n = 27).


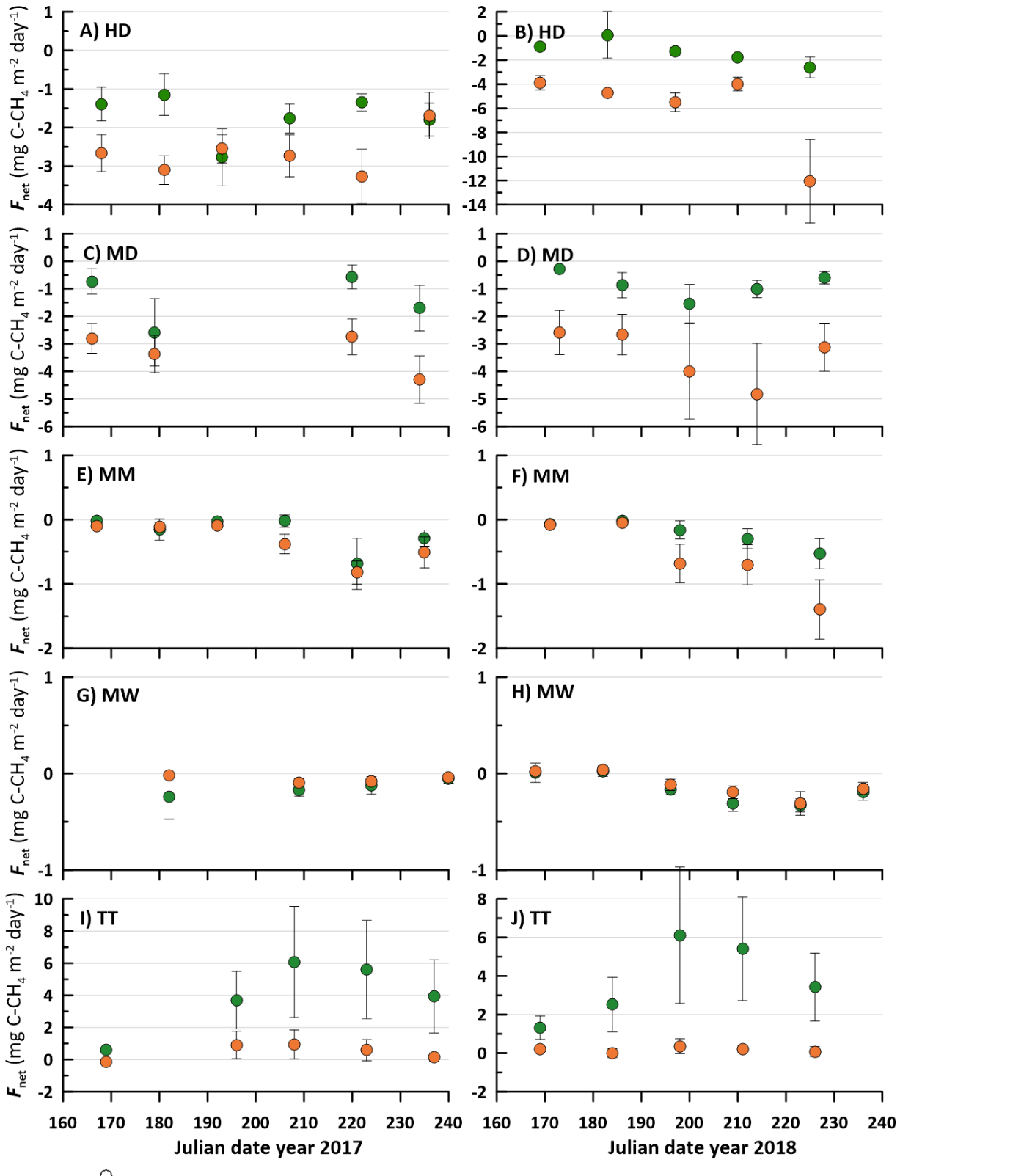


**Figure S2**. Average bi-weekly growing season *F*_net_ measurements (2017 and 2018) in the five investigated plant communities are shown with error bars indicating S.E. estimates.


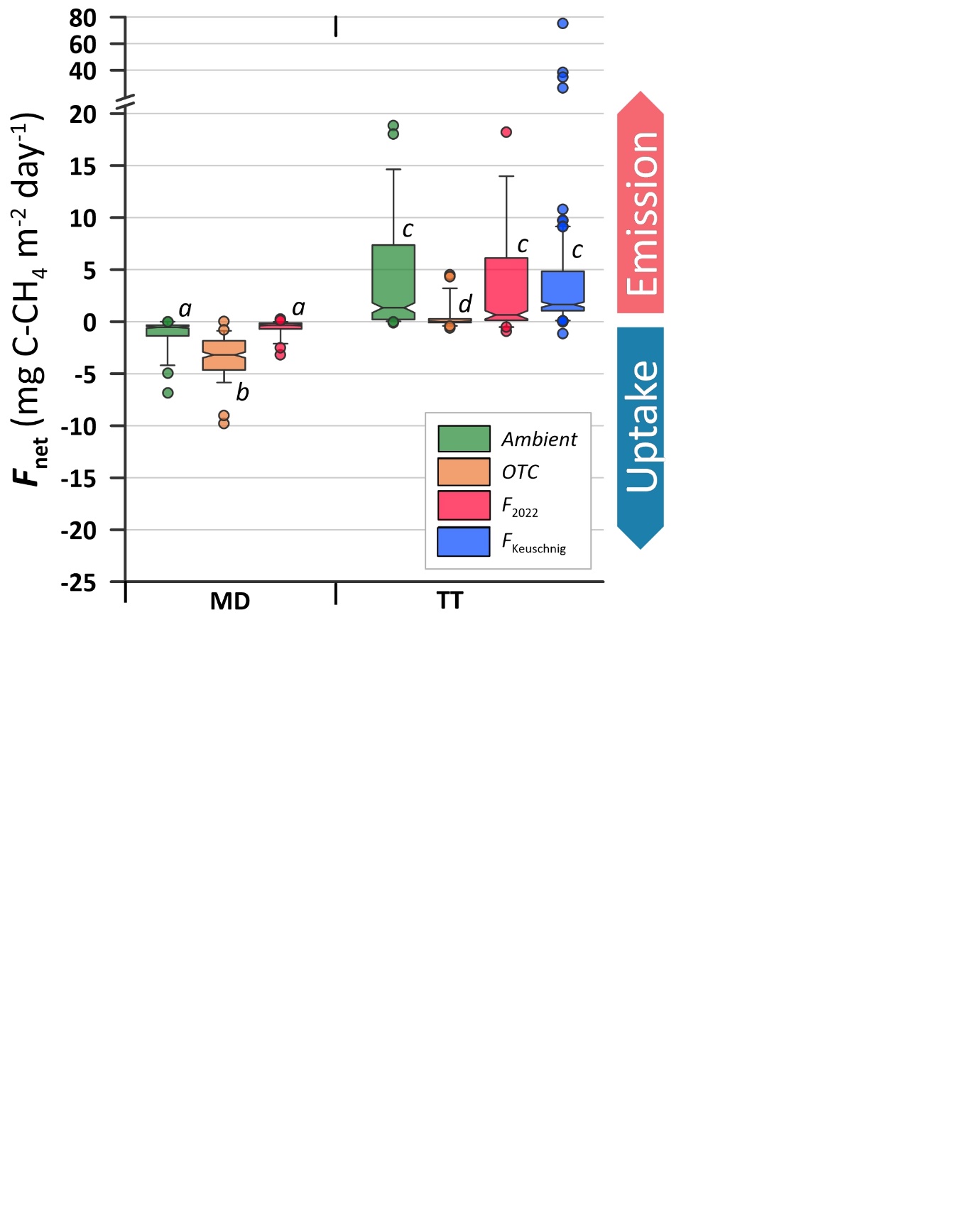


**Figure S3**. Summary of bi-weekly growing season CH_4_ fluxes included in this study (*Ambient* and *OTC*) and an additional survey from 2022 (*F*_2022_) in the Dry Meadow (MD) and the Tussock Tundra (TT) communities, along with an earlier study (*F*_Keuschnig_) from the TT using other ambient plots. Different lower-case letters indicate a significant difference among the ambient plots in each plant community (p < 0.05).


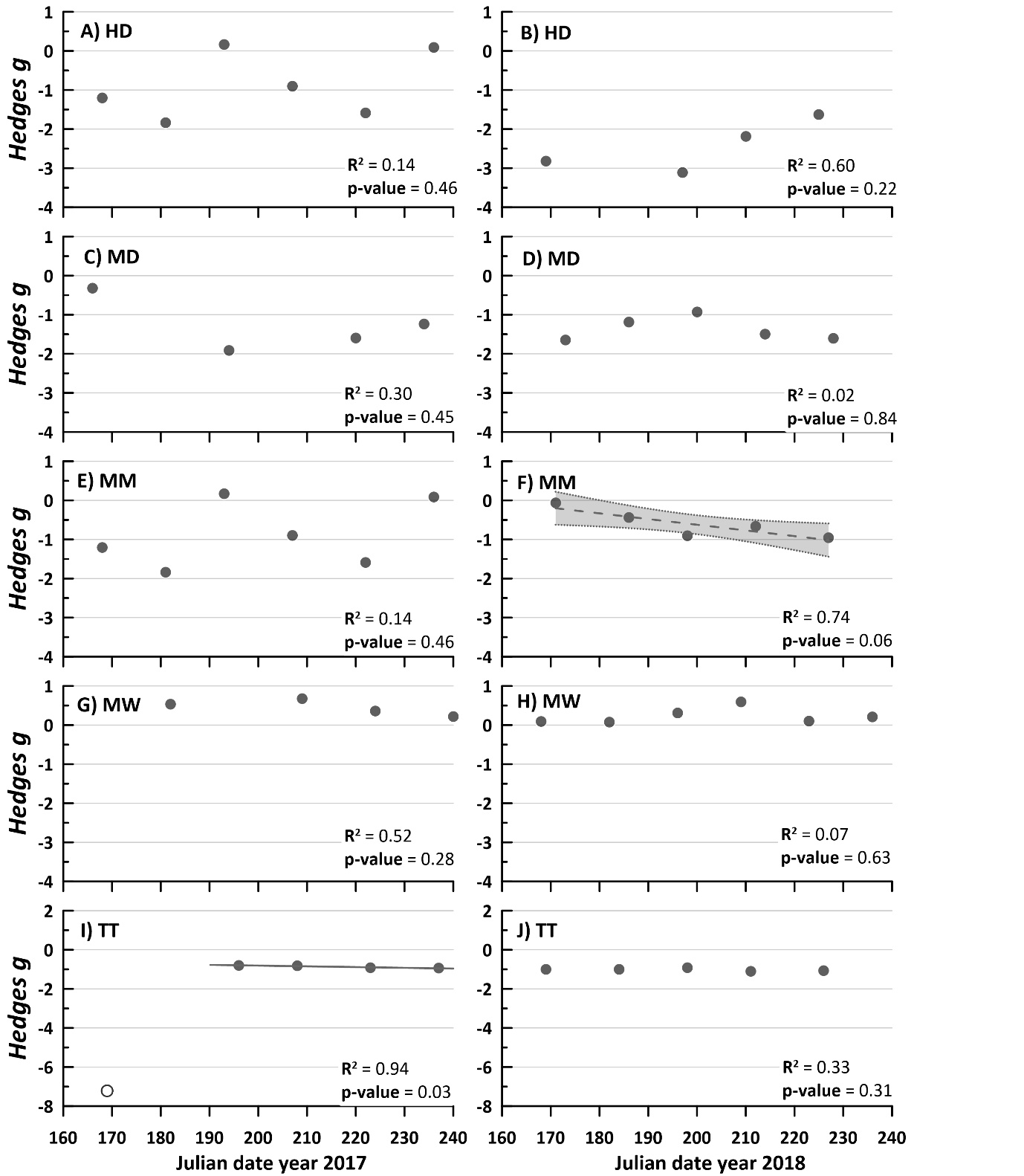


**Figure S4**. The progression of Hedges' g effect size over the seasons (2017 and 2018) for each plant community.


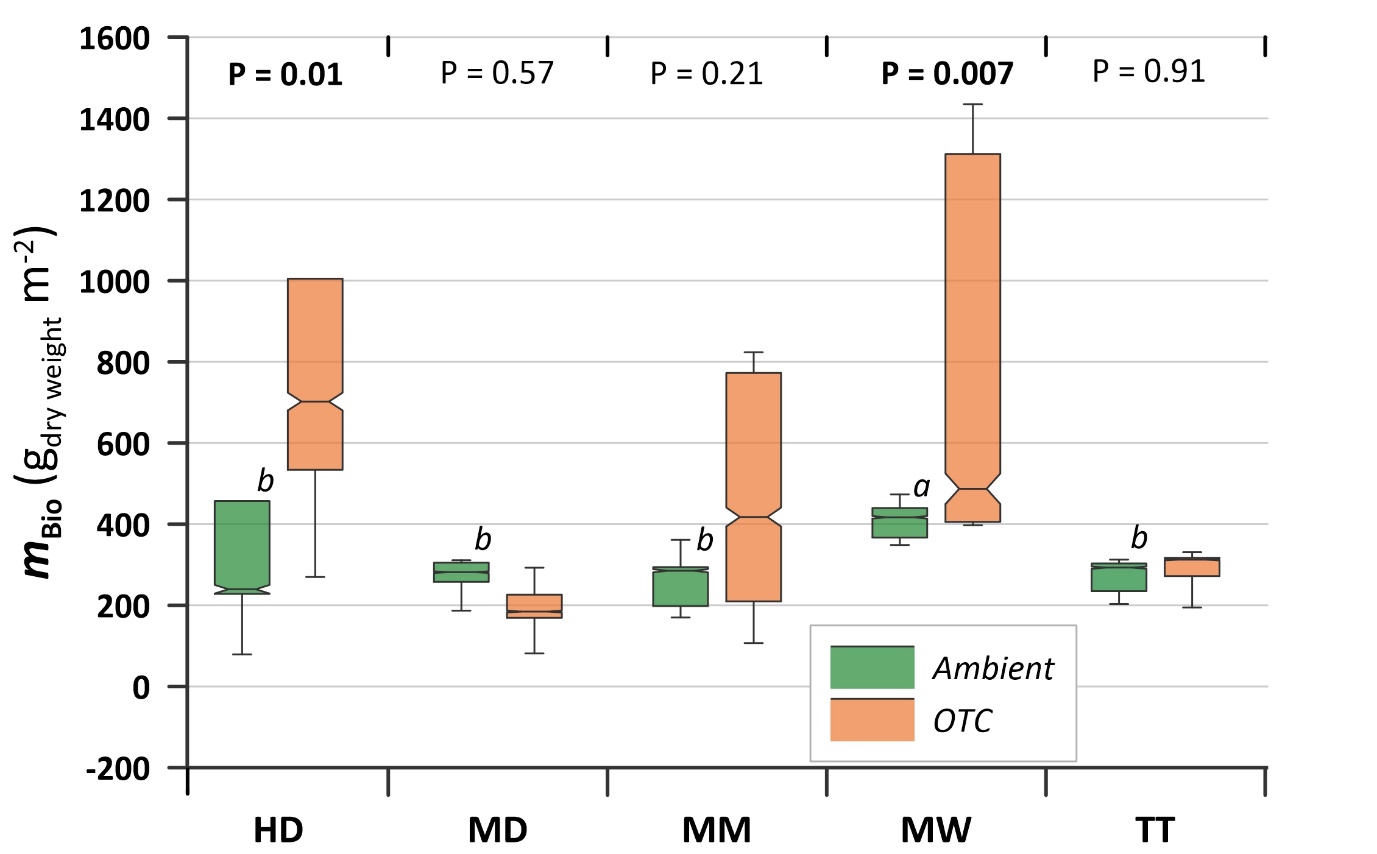


**Figure S5**. The estimated biomass of the field layer was calculated from the point-frame data (2016), plant trait data (2019 and 2020) and group-specific correlations as stated by Molau 2010. The significance between *Ambient* and *OTC* plots is given at the top of the plot, using a Welch Two-Sample t-test. The total *n* for each vegetation type and treatment is five for all communities except HD, where *n* = four. Different lower-case letters indicate a significant difference among the *Ambient* plots between plant communities (here, a threshold was set to p < 0.10 due to the low number of replicates).


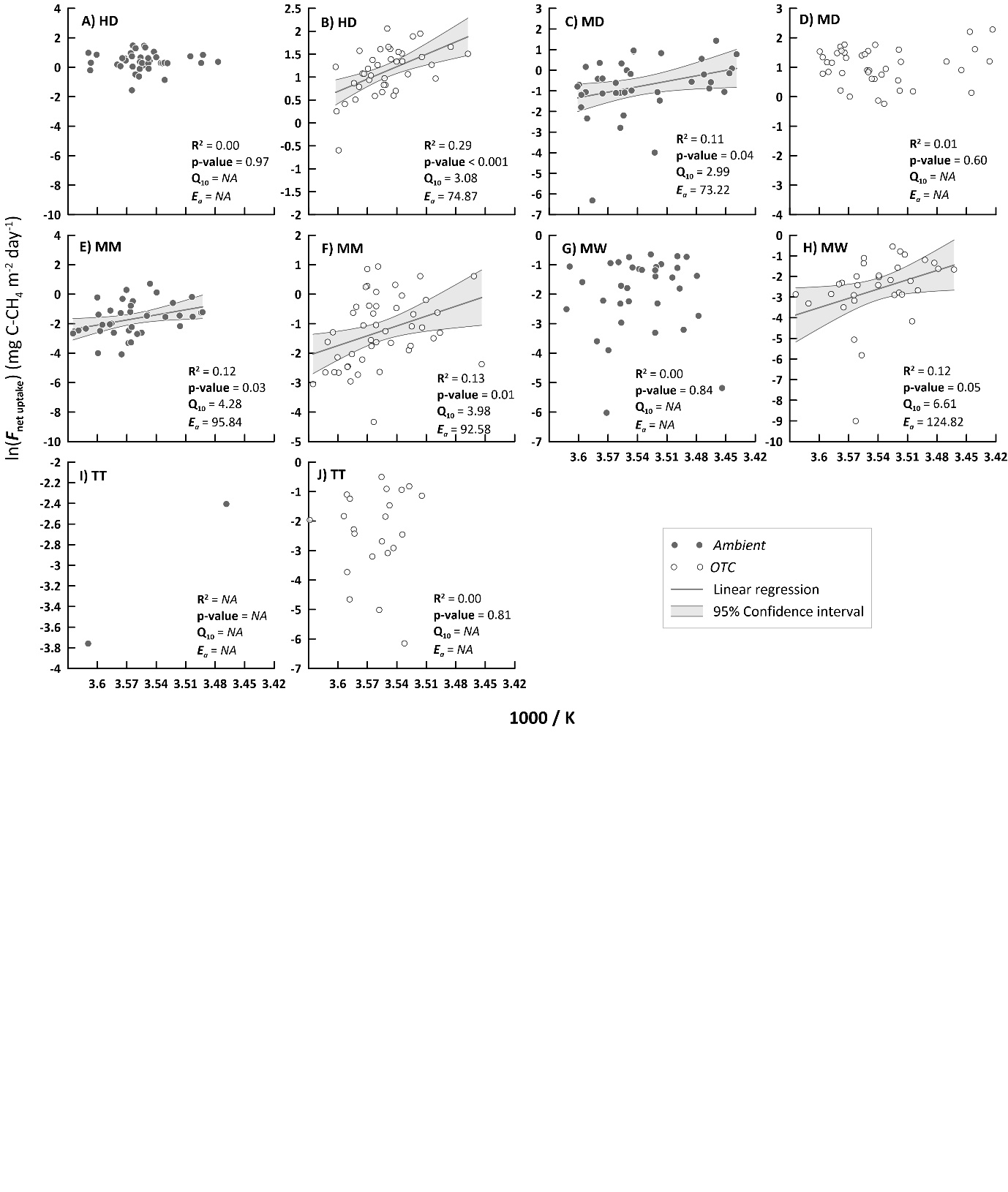


**Figure S6**. Arrhenius relationship between the natural logarithm of *F*_net_, when *F*_net_ was negative (uptake), and soil temperature (- 5cm). The estimated values for Q_10_ and *E*_a_ are only given for significant relationships.


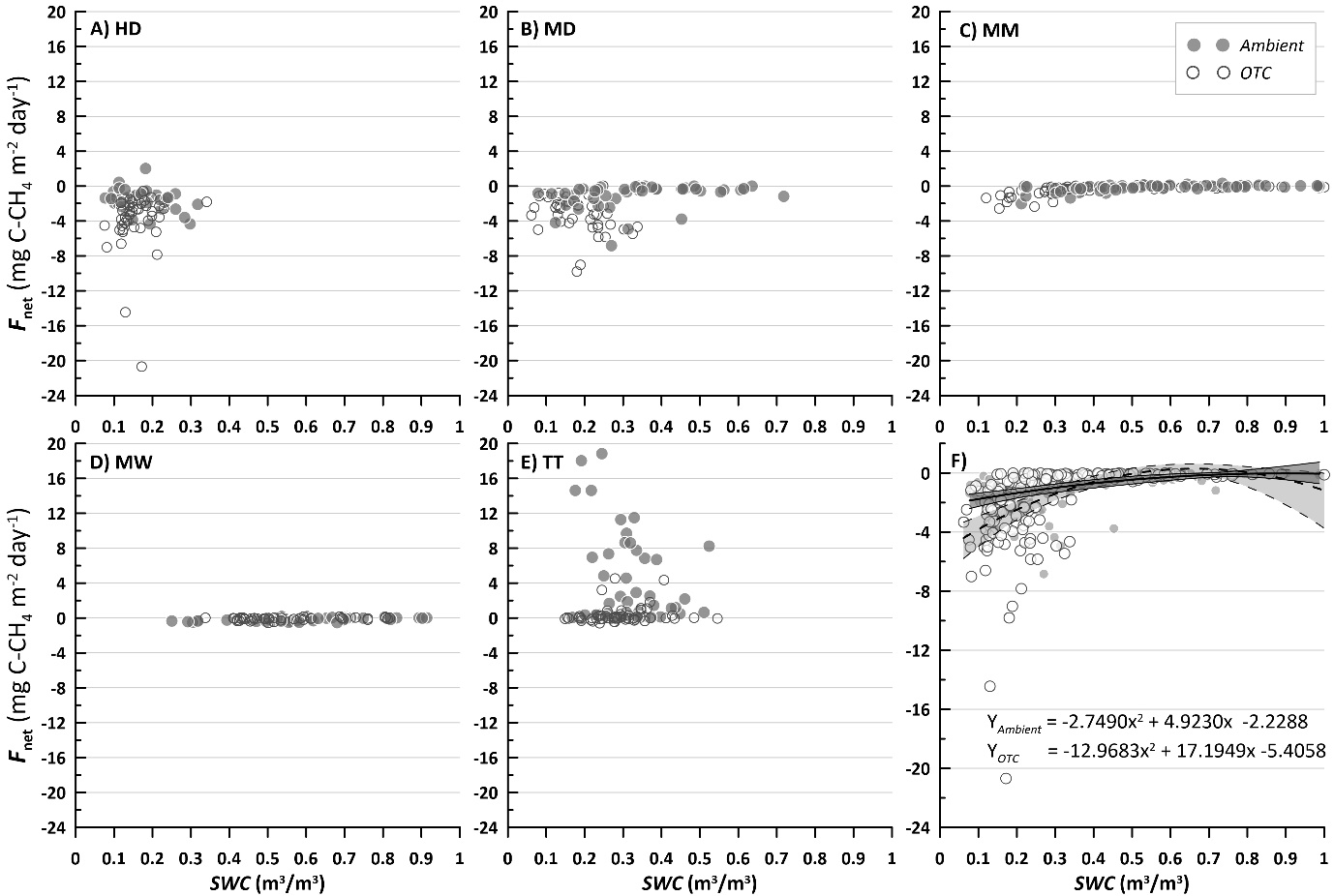


**Figure S7**. *F*_net_ as a function of soil water content, SWC, for each plant community and treatment: A) HD, B) MD, C) MM, D) MW and E) TT. F) gives the relationship between soil uptake of CH_4_ and soil water content, viewed as a second-order polynomial function according to D’Imperio et al. (2017) for all *Ambient* (soil lines) and *OTC* (broken lines) plots. The R^2^ for the *Ambient* and *OTC* relationships are 0.26 and 0.24, respectively, both with a P-value < 0.0001.
